# Response Prediction to Concurrent Chemoradiotherapy in Esophageal Squamous Cell Carcinoma Using Delta-Radiomics Based on Sequential Whole-Tumor ADC Map

**DOI:** 10.3389/fonc.2022.787489

**Published:** 2022-03-15

**Authors:** Dianzheng An, Qiang Cao, Na Su, Wanhu Li, Zhe Li, Yanxiao Liu, Yuxing Zhang, Baosheng Li

**Affiliations:** ^1^ Department of Radiation Oncology, Shandong Cancer Hospital Affiliated to Shandong University, Shandong University, Jinan, China; ^2^ Department of Radiation Oncology, Shandong Cancer Hospital and Institute, Shandong First Medical University and Shandong Academy of Medical Sciences, Jinan, China; ^3^ Department of Radiology, Shandong Cancer Hospital and Institute, Shandong First Medical University and Shandong Academy of Medical Sciences, Jinan, China; ^4^ Department of Imaging, Anyang Tumor Hospital, The Fourth Affiliated Hospital of Henan University of Science and Technology, Anyang, China

**Keywords:** esophageal squamous cell carcinoma, concurrent chemoradiotherapy, diffusion-weighted imaging, radiomics, treatment response

## Abstract

**Purpose:**

The purpose of this study was to investigate the association between the radiomics features (RFs) extracted from a whole-tumor ADC map during the early treatment course and response to concurrent chemoradiotherapy (cCRT) in patients with esophageal squamous cell carcinoma (ESCC).

**Methods:**

Patients with ESCC who received concurrent chemoradiotherapy were enrolled in two hospitals. Whole-tumor ADC values and RFs were extracted from sequential ADC maps before treatment, after the 5th radiation, and after the 10th radiation, and the changes of ADC values and RFs were calculated as the relative difference between different time points. RFs were selected and further imported to a support vector machine classifier for building a radiomics signature. Radiomics signatures were obtained from both RFs extracted from pretreatment images and three sets of delta-RFs. Prediction models for different responders based on clinical characteristics and radiomics signatures were built up with logistic regression.

**Results:**

Patients (n=76) from hospital 1 were randomly assigned to training (n=53) and internal testing set (n=23) in a ratio of 7 to 3. In addition, to further test the performance of the model, data from another institute (n=17) were assigned to the external testing set. Neither ADC values nor delta-ADC values were correlated with treatment response in the three sets. It showed a predictive effect to treatment response that the AUC values of the radiomics signature built from delta-RFs over the first 2 weeks were 0.824, 0.744, and 0.742 in the training, the internal testing, and the external testing set, respectively. Compared with the evaluated response, the performance of response prediction in the internal testing set was acceptable (*p* = 0.048).

**Conclusions:**

The ADC map-based delta-RFs during the early course of treatment were effective to predict the response to cCRT in patients with ESCC.

## Introduction

Worldwide, esophageal cancer ranks 7th in cancer incidence and 6th in mortality rate reported by the World Health Organization (1). Esophageal squamous cell carcinoma (ESCC) is one of the top ten characteristic tumors in China with a proportion over 90% in all esophageal cancer patients, while adenocarcinoma is dominant in the West (2). The 5-year survival rate for locally advanced ESCC is limited to 15-30%, and the prognosis varies from patients even with the same treatment (3, 4). Though concurrent chemoradiotherapy (cCRT) has become the prevalent treatment for local advanced ESCC, individual differences for therapeutic sensitivity still exist (5). It is challenging to accurately predict the sensitivity to cCRT and distinguish the discrepancy of ESCC to assist clinical decisions.

Response Evaluation Criteria in Solid Tumors (RECIST, version1.1) is the most widely used tool for assessing solid tumor response to nonsurgical treatment. It relies on imaging techniques, such as X-ray and computed tomography (CT), providing little information about the molecule, cell, histopathology, and biology (6). Compared to these imaging modalities, magnetic resonance imaging (MRI) is able to precisely depict the histopathological layers of the esophageal wall in an *ex vivo* evaluation (7). Notably, diffusion-weighted imaging (DWI) with physiological information reflecting water diffusion properties of tissues is potentially practical to monitor tumor response. The apparent diffusion coefficient (ADC) and the change of ADC (delta-ADC/ΔADC) generated from DW-MRI showed a correlation with tumor response to treatment (8–10). Nevertheless, the utilization of the ADC value as an imaging biomarker is still controversial (11–13).

With the development of computerized image processing technology during the past decades, quantitative imaging analysis is becoming more and more popular in radiology (14). Radiomics, which extracts numeric radiologic data from medical imaging, quantitatively describes the shape, intensity, texture, and other features of target structures (15). It has been reported that various radiomics features (RFs) are associated with tumor genes, pathology, and outcome of treatment in different tumors (16–20). Furthermore, the change of RFs between pretreatment images and images of other time points during or after treatment, referred to as delta-radiomics, has been investigated in lung cancer and rectal cancer as promising prognostic factors (21, 22). Theoretically, delta-RFs (ΔRFs) reflect much detailed information of changes induced by chemotherapy, radiation, and immunotherapy throughout treatment rather than just at pretreatment.

It is assumed that radiomics analysis of the ADC map is helpful to predict early treatment response to cCRT of ESCC patients. Aiming to predict response to concurrent CRT in patients with ESCC, this study developed radiomics models based on RFs and ΔRFs during early treatment. In addition, the prediction value of ADC and ΔADC values were also analyzed.

## Materials and Methods

### Study Population

All protocols of this prospective and observational study were approved by the Institutional Review Board (Project ID: 201404005). Patients from Shandong Cancer Hospital and Institute were randomly assigned to the training set and the internal testing set in a ratio of 7:3 to ensure an adequate sample size. The external testing set was enrolled in Anyang Tumor Hospital.

All methods were carried out following relevant guidelines and regulations. All patients provided written informed consent before enrollment. Inclusion criteria were as follows: (a) ESCC histologically proven by endoscopic biopsy; (b) no contraindications to MR examination; (c) clinical stage T3 or T4 defined on endoscopic ultrasonography, diagnostic CT scans, or ^18^fluorodeoxyglucose positron emission tomography/computed tomography (^18^F-FDG PET/CT) scan; (d) no prior anticancer treatment; (e) no distant metastasis except lymph nodes; (f) no other primary tumor. Clinical staging was based on the tumor-node-metastasis classification of malignant tumors (UICC, Version 8th). All patients were treated with concurrent CRT. The daily fractional dose of radiotherapy was 1.8–2.0 Gy, administered 5 days a week, and the total dose was 50.4–66.0 Gy with 6–15 MV X-rays performed by intensity-modulated radiation therapy. Before the early treatment response evaluated, all patients accepted two cycles of chemotherapy concurrently during radiotherapy every 3 weeks. One of the following proposals of chemotherapy would be adopted: 1) an intravenous injection of paclitaxel (150 mg/m^2^) on day 1 and cisplatin (75 mg/m^2^) on days 1–3; and 2) S-1 administered orally twice daily at 80 mg/m^2^ for 2 weeks and cisplatin (75 mg/m^2^) on days 1–3.

### Response Assessment

The response was assessed according to RECIST 1.1 (6). Upper gastrointestinal endoscopy, chest and abdomen CT, or whole-body ^18^F-FDG PET/CT was performed 2–3 months after treatment finished to compare with the pretreatment images. The definition of chemoradiotherapy sensitivity was as follows: the sensitive group comprised patients with complete response (CR) and partial response (PR); the resistant group consisted of patients with stable disease (SD) and progression disease (PD).

### Sequential MRI Acquisition

**Figure 1:**
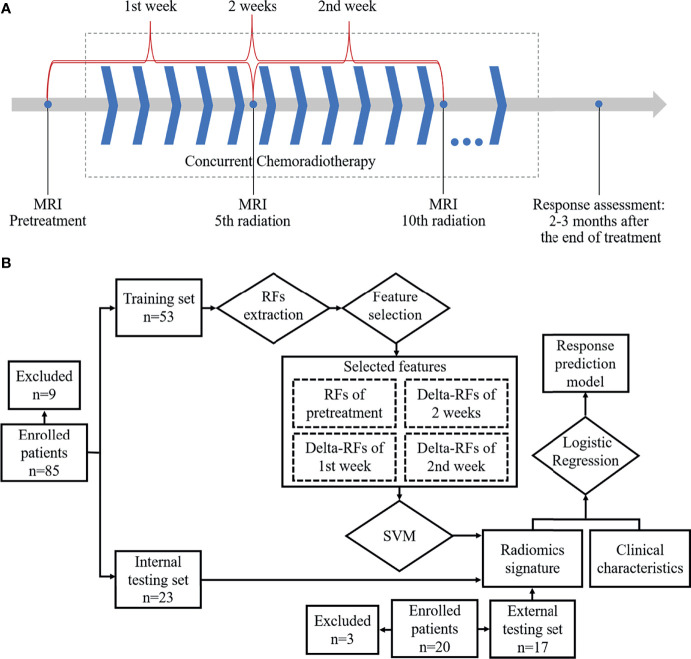
Workflow: **(A)** Magnetic resonance imaging acquisition schema and time ranges of the change of radiomics features. **(B)** Workflow for building radiomics signature-based prediction model.

MRI examination with DWI and T2-weighted imaging (T2WI) in the axial orientation was sequentially performed at about 1–3 days before treatment, 5^th^ fraction of radiation completed (5f), and 10^th^ fraction of radiation completed (10f) as shown in **Figure 1A**. All patients were scanned using a 3.0 T MRI system (Achieva, Philips Medical Systems, Netherlands; Ingenia, Philips Medical Systems, Netherlands) with body phased-array coil anterior and spine array coil posterior. T2WI was performed by using a two-dimensional fluid-attenuated inversion recovery sequence (repetition time/echo time, 1,277 ms/70 ms; flip angle, 140°; bandwidth, 315 kHz; section thickness, 4 mm; slice gap, 1 mm; field of view, 350 × 350mm; matrix, 384 × 276; no. of slices, 24–36; imaging time, 5 minutes). DWI was performed by using a single-shot echo-planar imaging (repetition time/echo time, 1,244.7 ms/47.9 ms; flip angle, 140°; bandwidth, 260 kHz; section thickness, 4 mm; slice gap, 1 mm; field of view, 360 × 360 to 400 × 400 mm; matrix, 128 × 128; no. of slices, 24–36; imaging time, 5 minutes). The number of signal averaged (NSA) was 1. Diffusion gradients were applied in three orthogonal directions by different diffusion weightings (*b*-values) of 0, 300, and 600 s/mm^2^. Especially, *b* = 600 s/mm^2^ was assigned a NSA of 4 to improve the signal-to-noise ratio. Respiratory triggering was used.

The ADC map was generated automatically at the MR workspace (Philips Medical Systems Extended MR workspace, Netherlands) from DWI, which was overlayed and averaged in three directions with the *b*-value of 0 and 600 s/mm^2^.

### ADC Value and Extraction of Original RFs

**Figure 2:**
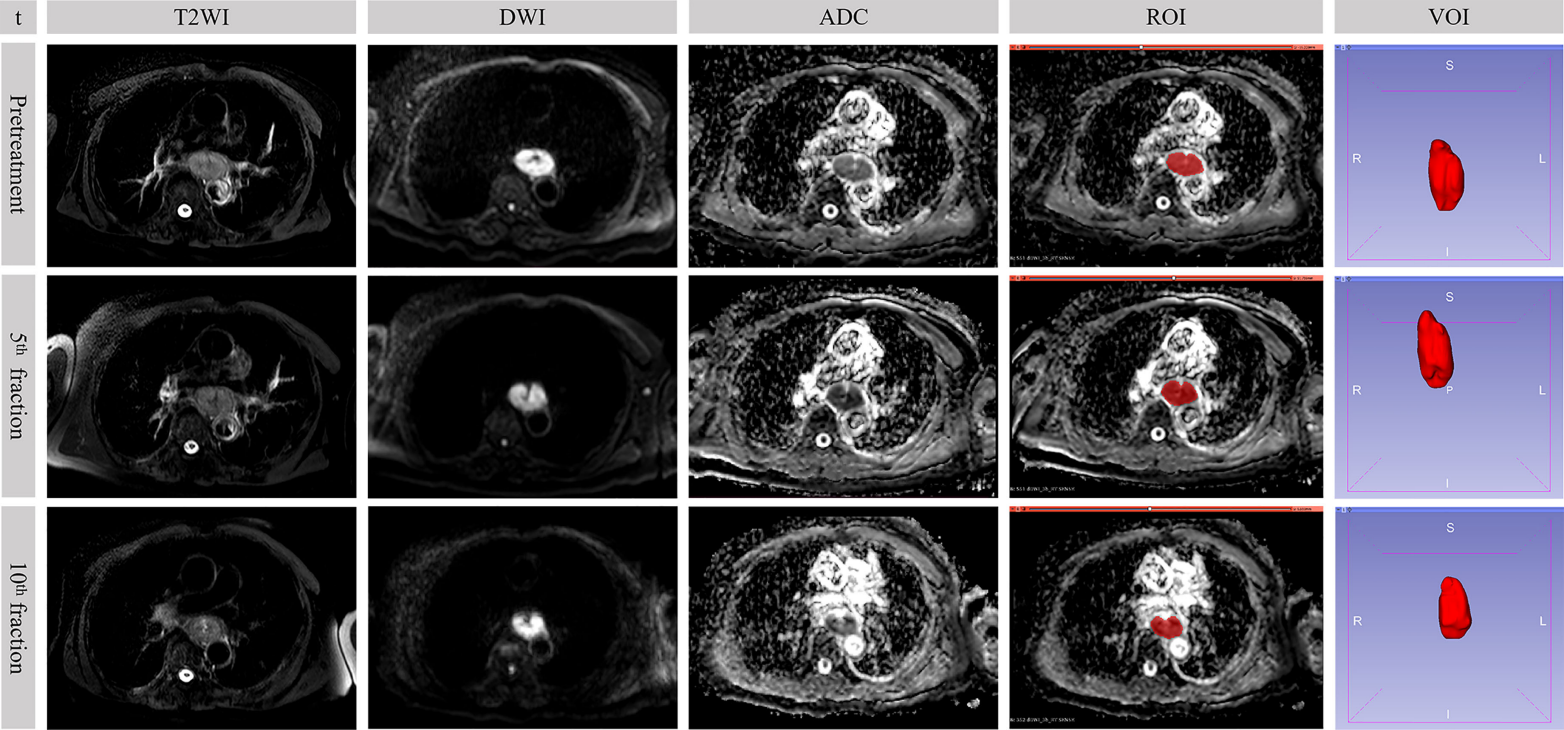
Sequential MR images in a 73-year-old male who underwent concurrent chemoradiotherapy with partial response. The value of *b*-factor of DWI in the figure was 600 s/mm^2^. t, time point; DWI, diffusion-weighted imaging; ADC, apparent diffusion coefficient; ROI, region of interest; VOI, volume of interest.

The location of the primary tumor was firstly identified on DWI (*b* = 600 s/mm^2^) as areas of high signal (**Figure 2**). Although it was not outlined on DWI, it assisted in accurately finding the corresponding location of the tumor in the ADC map. Regions of interest (ROIs) were manually drawn on the ADC map *via* a segmentation editor in the 3D Slicer software (version 4.10.0, http://www.slicer.org) (23). The definition of ROI was the region of the tumor that was of low signal in the ADC map but excluding the lumen. Every slice containing the tumor was outlined. These ROIs constituted a volume of interest (VOI) with the entire tumor visualized on the ADC map as showed in **Figure 2**. The mean tumor ADC value was calculated by averaging the measured ADC values of the VOI. For whole-tumor radiomics feature, the Slicer Radiomics, which was based on PyRadiomics program (Revision 2.2.0) in the 3D Slicer software, automatically extracted whole-tumor RFs from the three-dimensional reconstructed VOI (24). In our practice, resampled voxel size, LoG kernel sizes, and bin width were respectively set to 1, 1, and 25. In total, 851 RFs were extracted including 18 first-order statistics features, 14 shape-based (2D and 3D) features, 24 gray-level co-occurrence matrix (GLCM) features, 16 gray-level run length matrix (GLRLM) features, 16 gray-level size zone matrix (GLSZM) features, 5 neighboring gray tone difference matrix (NGTDM) features, 14 gray-level dependence matrix (GLDM) features, and 744 wavelet-based features. Detailed classification of the extracted RFs was summarized in **Supplementary Table 1**. In addition, the RFs mentioned in our study were consistent with the definition of the image biomarker standardization initiative (25).

### RF Selection

Selection procedures were performed on the pretreatment images with three steps.

Because DWI is susceptible to field inhomogeneities, large amounts of magnetization artifacts in the chest area, and patient movement, two consecutive DWI acquisitions of the same patient may give images with slightly different imaging characteristics, which may affect radiomics analysis. Thus, the first is to evaluate the repeatability of the radiomics features between two ADC maps in a short time by the intraclass correlation coefficient (ICC) method. Five extra patients with ESCC were enrolled before the first treatment in Shandong Cancer Hospital and Institute (**Supplementary Table 2**) who were not included in the training and internal testing sets. The interval between the two consecutive DWI scans was 5 minutes (**Supplementary Figure 1**). VOIs were delineated in the ADC map by an experienced radiologist (WL, with 20 years of experience in MRI), then the VOI was duplicated to the other ADC map from the same patient. The reliability coefficient of RFs was supposed to be higher than 0.75.

Secondly, to select RFs with high interobserver reproducibility, VOIs were delineated by two experienced radiologists (WL, appointed as reader 1; NS, appointed as reader 2 with 11 years of experience in MRI) who were blind to clinical information, treatment plan, and response about patients. Bland–Altman analysis was used to test the interobserver reproducibility of the RFs from the VOI delineated by different radiologists. The differences between the same parameters extracted from two readers’ delineation were plotted against their average and reported as a percentage. The lower and upper reproducibility limits were calculated as ± 1.96 standard deviations. In our case, the mean and standard deviation of the differences were supposed to be less than 5% and 10%, respectively, as stated in related work (26, 27).

Thirdly, to reduce the number of features and select the most significant features correlated with treatment response, the minimum redundancy maximum relevance (mRMR) algorithm was used to identify and rank the top 30 features. Finally, the selected RFs were applied in the training, internal, and external testing sets from the delineation of reader 1.

### Change of the Mean of ADC Values and RFs

The changes of the whole-tumor ADC value between images of different time points were respectively calculated as follows:


(Eq.1) $$\Delta ADC_{1st\;week}=\frac{ADC_{5f}-ADC_{pretreatment}}{ADC_{pretreatment}}$$

(Eq.2) $$\Delta ADC_{2nd\;week}=\frac{ADC_{10f}-ADC_{5f}}{ADC_{5f}}$$

(Eq.3) $$\Delta ADC_{2\;weeks}=\frac{ADC_{10f}-ADC_{pretreatment}}{ADC_{pretreatment}}$$

where ΔADC_1st week_, ΔADC_2nd week_, and ΔADC_2 weeks_ denoted the change of the ADC value within the 1st week, the 2nd week, and the first two weeks, respectively. Similarly, ADC_pretreatment_, ADC_5f_, and ADC_10f_ denoted the ADC value of images acquired before treatment, after the 5th radiation, and after the 10th radiation, respectively.

The changes of the selected RFs were defined as the relative difference between each measurement as follows:


(Eq.4) $$\Delta RF_{1st\;week}=\frac{RF_{5f}-RF_{pretreatment}}{RF_{pretreatment}}$$

(Eq.5) $$\Delta RF_{2nd\;week}=\frac{RF_{10f}-RF_{5f}}{RF_{5f}}$$

(Eq.6) $$\Delta RF_{2\;weeks}=\frac{RF_{10f}-RF_{pretreatment}}{RF_{pretreatment}}$$

where ΔRF_1st week_, ΔRF_2nd week_, and ΔRF_2 weeks_ denoted the change of RFs within the 1st week, 2nd week, and first two weeks, and RF_pretreatment_, RF_5f_, and RF_10f_ denoted the RFs in images acquired before treatment, after the 5th radiation, and after the 10th radiation, respectively.

### Construction of Prediction Model Based on Radiomics Signature

As shown in **Figure 1B**, RF_pretreatment_, ΔRF_1st week_, ΔRF_2nd week_, and ΔRF_2 weeks_ were used to train a support vector machine (SVM) classifier in the training set, and then a radiomics signature was built by the trained classifier and evaluated in the internal and external testing sets. The cost‐based SVM classifier, which used the radial basis function kernel and a cost coefficient of 0.001, was trained by RFs with the 10-fold cross-validation. The SVM classifier transformed the feature space to a high-dimensional space where a separating hyperplane maximized the margin between classes. For each patient, the SVM classifier generated a radiomics signature for evaluating predictions of treatment response. The receiver operating characteristic (ROC) curves of obtained radiomics signatures in the training, internal, and external testing sets were analyzed and then tested by the DeLong test. The chi-square test was used to compare the predicted responses, which were corresponding to radiomics signatures in the internal testing with the signatures’ ground truth, i.e., treatment response evaluated by RECIST1.1. Afterward, the eligible radiomics signatures together with clinical characteristics were imported to a multivariate logistic regression-based prediction model to predict the sensitivity to treatment. Furthermore, the performance of radiomics signatures, the ADC value, clinical characteristics, and the regression model was assessed by the ROC curve, respectively. The area under the curve (AUC) was calculated, and the optimal Youden’s index was determined.

### Statistical Analysis

Statistical analysis was performed using the R software (version 3.6.1, http://www.R-project.org). The ICC method, Bland–Altman method, mRMR approach, SVM classifier, logistic regression analysis, and ROC curve analysis were implemented based on the R packages “irr”, “BlandAltmanLeh”, “mRMRe”, “e1071”, and “pROC.” Chi-square test or t-test was used to examine the correlations between clinical features and treatment response. The differences in ADC and ΔADC values were compared between the sensitive group and the resistant group by Mann–Whitney U test. *P*-value < 0.05 was considered as an indicator of the statistically significant difference.

## Results

### Clinical Characteristics

In Shandong Cancer Hospital and Institute, eighty-five consecutive patients with ESCC were enrolled. Nine patients were excluded, given that the DWI of 6 patients were deformed or had high noise and 3 patients’ radiotherapy was interrupted. Finally, 76 patients were enrolled in the study during the period of enrollment from June 2014 to September 2019. Fifty-four of the 76 patients experienced clinical PR, 2 with CR and 20 with SD. Patients (n=76) were randomly assigned to training (n=53) and internal testing sets (n=23) in a ratio of 7 to 3. There was no significant difference in clinical characteristics between the training and internal testing sets (sex, age, tumor location, clinical T stage, lymph node status, and radiation dose) and treatment response in both sets (**Table 1**). In Anyang Tumor Hospital, twenty consecutive patients with ESCC were enrolled during the period of enrollment from April 2017 to May 2019. Three patients were excluded, given that 3 patients’ radiotherapy was interrupted. Finally, 17 patients were enrolled in the external testing set. Eleven of the 17 patients experienced clinical PR and 6 with SD. Clinical characteristics are presented in **Table 1**.

**Table 1:** Patients and tumor characteristics association with treatment response in the training, internal, and, external testing sets.

Characteristics	Training set (n=53)		*p*	Internal testing set (n=23)		*p*	*p* ^†^	External testing set (n=17)		p
	Sensitive group (n=36)	Resistant group (n=17)		Sensitive group (n=18)	Resistant group (n=5)		0.361	Sensitive group (n=11)	Resistant group (n=6)	
Sex			0.821			0.048	0.793			1.000
Male	28	12		16	3			6	3	
Female	8	5		2	2			5	3	
Age (year)			0.412			0.446	0.675			0.660
mean ± sd	63.1 ± 8.14	65.2 ± 10.3		63.9 ± 5.6	59.0 ± 10.7			62.2 ± 8.2	64.5 ± 6.1	
range	43-78	44-80		51-72	39-70			51-72	39-70	
T-stage			0.730			0.576	0.602			0.515
T3	25	11		14	3			10	4	
T4	11	6		4	2			1	2	
LN Status			0.647			1.000	0.834			1.000
N-	7	5		3	1			4	2	
N+	29	12		15	4			7	4	
Location			0.023			0.662	1.000			0.043
Cervival	0	4		1	1			1	3	
Upper thoracic	15	7		7	2			6	2	
Middle thoracic	14	3		6	2			4	0	
Lower thoracic	7	3		4	0			0	1	
Dose (Gy)			0.234			1.000	0.823			1.000
<55	19	6		9	2			9	2	
≥55	17	11		8	3			8	3	

LN, lymph node.

^†^ Difference between the training set and the internal testing set in characteristics of patients and tumor.

*P < 0.05, statistically significant.

We measured the voxel volume of the whole-tumor ADC map to substitute the tumor volume and the maximum 3D diameter and their changes between each time point. **Supplementary Tables 3** **,** **4** show that these parameters had no relationship with treatment response.

### Association of Sequential ADC Value and Treatment Response

The mean values of ADC_pretreatment_, ADC_5f_, and ADC_10f_ of all patients are shown in **Table 2**. All of the ADC values and relative changes during the first two weeks showed no significant difference between the sensitive and resistant groups in the training and internal testing sets. In the external testing set, ADC in the 5^th^ radiation showed association with treatment response (*p* = 0.048). We noticed that the ADC value was gradually higher than before treatment as the treatment progressed.

**Table 2:** Association between ADC values or ΔADC values and treatment response in the training, internal, and external testing sets.

Set	Time point	ADC value (10^-3^ mm2/s)		*p*	Time range	ΔADC		p
		Sensitive group	Resistant group			Sensitive group	Resistant group	
**Training set**	Pre-treatment	1.695 ± 0.687	1.644 ± 0.626	0.746	1st week	0.200 ± 0.415	0.135 ± 0.293	0.391
	5^th^ radiation	1.723 ± 0.612	2.067 ± 0.875	0.253	2nd week	-0.160 ± 0.218	-0.175 ± 0.286	0.819
	10^th^ radiation	2.057 ± 0.720	2.118 ± 0.697	0.381	2 weeks	0.275 ± 0.370	0.255 ± 0.400	0.746
**Internal testing set**	Pre-treatment	1.556 ± 0.599	1.466 ± 0.946	0.363	1st week	0.177 ± 0.444	0.351 ± 0.396	0.491
	5^th^ radiation	1.847 ± 0.624	1.893 ± 0.990	0.914	2nd week	-0.152 ± 0.239	0.126 ± 0.630	0.538
	10^th^ radiation	2.145 ± 0.831	1.973 ± 0.924	0.691	2 weeks	0.405 ± 0.595	0.538 ± 0.692	0.745
**External testing set**	Pre-treatment	1.948 ± 0.247	1.818 ± 0.388	0.149	1st week	0.065 ± 0.037	0.042 ± 0.0447	0.216
	5^th^ radiation	2.084 ± 0.237	1.787 ± 0.422	0.048^*^	2nd week	0.119 ± 0.117	0.145 ± 0.113	0.591
	10^th^ radiation	2.387 ± 0.263	2.078 ± 0.283	0.078	2 weeks	0.177 ± 0.110	0.179 ± 0.127	1.000

ADC, apparent diffusion coefficient.

*P < 0.05, statistically significant.

### Generation and Validation of the Radiomics Signature

**Figure 3:**
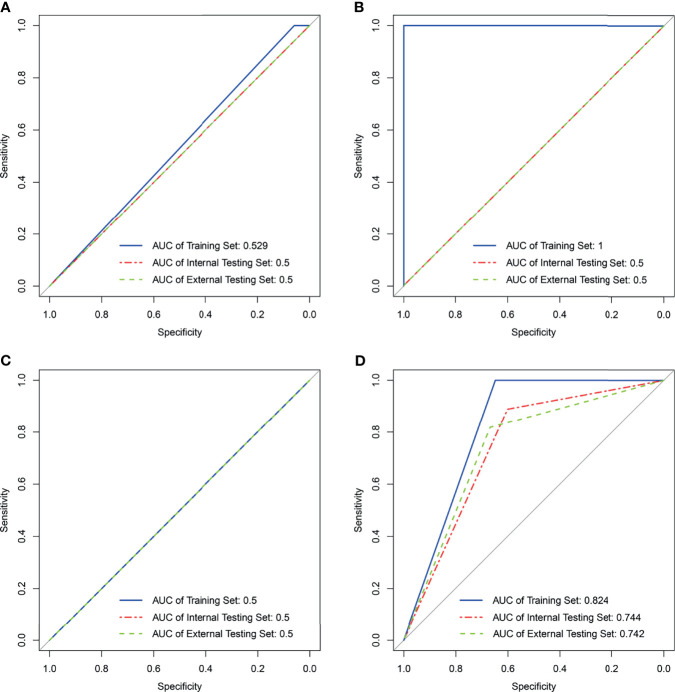
Results of support vector machine classifier in the training, internal, and external testing sets with four kinds of radiomics features. **(A)** Result of SVM generating radiomics signature_pretreatment_. **(B)** Result of SVM generating radiomics signature_1st week_. **(C)** Result of SVM generating radiomics signature_2nd week_. **(D)** Result of SVM generating radiomics signature_2 weeks_. NA in **(B, C)** means not applicable.

A total of 560 RFs (**Supplementary Table 5**) were selected by the ICC method from two consecutive whole-tumor ADC map acquisitions in the same patient. Afterward, 224 RFs (**Supplementary Table 6**) were selected from pretreatment images in the training set *via* the Bland–Altman method, and 30 RFs (**Supplementary Table 7**) were finally chosen out according to mRMR. Most of these RFs were calculated to measure the local homogeneity of the image. To predict good and bad responder, four radiomic signatures based on an SVM classifier were respectively built with RF_pretreatment_, ΔRF_1st week_, ΔRF_2nd week_, and ΔRF_2 weeks_. The effectiveness of these 4 radiomics signatures to classify the sensitive group versus the resistant group is shown in **Figure 3**. Only the radiomics signature based on ΔRF_2 weeks_, denoted as R-Signature_2 weeks_, discriminated treatment response with an AUC value higher than 0.5 in the training set [AUC = 0.824, 95% confidence interval (CI): 0.679–0.968], the internal testing set (AUC = 0.744, 95% CI: 0.465–1.0), and the external testing set (AUC = 0.742, 95% CI: 0.478–0.919). No difference was found between the training and internal testing sets according to the results of the DeLong test (*p* = 0.580), nor between the training and external testing sets (*p* = 0.526). Compared with the evaluated response, the performance of response prediction in internal testing was acceptable (*p* = 0.048). The chi-square test of prediction results between R-Signature_2 weeks_ in the training set and the internal testing set achieved a *p*-value of 1.000, which implied that the distribution of R-Signature_2 weeks_ between the two sets was not statistically significantly different.

### Combining Model Analysis

**Figure 4:**
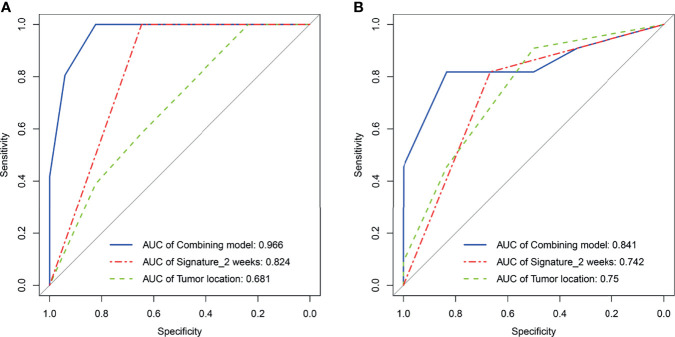
Receiver operating characteristic curves analysis for tumor location, radiomics signature_2 weeks_, and regression model, respectively, in the training set **(A)** and the external testing set **(B)**.

As elaborated in **Supplementary Table 8**, in the training set, the outputs of univariate logistics regression showed that tumor location and R-Signature_2 weeks_ were separately associated with treatment response. We performed the tumor location and R-signature_2 weeks_ to the prediction model of treatment response by multivariate logistics regression (**Supplementary Table 9**) in the three sets. R-Signature_2 weeks_ was identified as an independent factor predicting treatment response in multivariate analysis in the three sets. **Figure 4** shows the results of ROC analyses on the tumor location, R-Signature_2 weeks_, and the regression model in the prediction of treatment response in the training, internal, and external testing sets. **Table 3** shows the effectiveness of our proposed regression model in discriminating the sensitive and resistant groups.

**Table 3:** Performance of radiomics signatures and models for predicting treatment response in the training, internal, and external testing sets.

Set	Predictor	Sensitivity (%)	Specificity (%)	Accuracy (%)	PPV (%)	NPV (%)	*p*	AUC (95% CI)
	Tumor location	100.0	23.5	75.5	73.5	100.0	0.046	0.681 (0.504-0.773)
**Training** **set**	R-Signature_2 weeks_	100.0	64.7	88.7	85.7	100.0	<0.0001	0.824 (0.694-0.915)
	Tumor location+ R-Signature_2 weeks_	100.0	82.4	94.3	92.3	100.0	<0.0001	0.966 (0.875-0.996)
**Internal testing** set^*^	Tumor location	22.2	100.0	39.1	100.0	26.3	0.256	0.650 (0.425-0.835)
	R-Signature_2 weeks_	88.9	60.0	82.6	88.9	60.0	0.044	0.744 (0.522-0.901)
	Tumor location+ R-Signature_2 weeks_	–	–	–	–	–	–	–
**External testing set**	Tumor location	90.9	50	76.5	75.0	75.0	0.0499	0.674 (0.486-0.924)
	R-Signature_2 weeks_	81.8	66.7	76.5	81.8	66.7	0.0465	0.742 (0.478-0.919)
	Tumor location+ R-Signature_2 weeks_	81.8	83.3	82.3	90.0	71.4	0.027	0.841 (0.586-0.970)

^*^Tumor location was showed a p-value of 0.256, which means the model was not established.

PPV, positive predictive value; NPV, negative predictive value; AUC, area under curve; CI, confidence interval.

*P < 0.05, statistically significant.

## Discussion

In this study, a radiomics signature was developed that incorporated the change of RFs based on the whole-tumor ADC map for predicting treatment response to concurrent CRT in patients with ESCC. Compared with pretreatment, our study showed that the radiomics signature based on ΔRF_2 weeks_ was able to predict the treatment response for evaluations in the following 2–3 months.

DWI-MRI was widely and routinely performed in kinds of tumors for diagnosis and treatment assessment. As designed in our study, ADC values were obtained at pretreatment, after the 5th radiation, and after the 10th radiation. However, the mean value of pretreatment ADC was not significantly different between sensitive and resistant groups, which was consistent with the studies of Kozumi in patients with ESCC undergoing concurrent CRT (11). In fact, results of prediction of treatment response using ADC or ΔADC were not consistent among previous studies, or even the opposite (8–10, 12). In our study, ADC_5f_ in the external testing showed association with treatment response, which was different from those in the training and internal testing sets. Since the diffusion movement of water molecules in the tissue was closely related to the distribution of the intratumoral structure, the ADC value tended to be affected by many factors, such as cell density, nuclear area, nuclear–cytoplasmic ratio, tumor angiogenesis, and proteins (28–30). In addition, tissue edema commonly occurred during CRT because of changes in blood perfusion, cell death, and blocked lymphatic drainage induced by radiation and cytotoxicity, which further caused the change of the ADC value (31). We observed that the ADC value increased in both the sensitive group and the resistance group, but only one set of positive results was obtained in the external testing set. Thus, ADC_pretreatment_ and the changes during the first ten radiations were limited in predicting the treatment response.

The mean value of ADC and ΔADC reflected the average level of the Brownian motion of water molecules and a rough change in the whole tumor, while radiomics offered more heterogeneity information involving the above molecular and pathologic characteristics in different regions. The rise of radiomics using quantitative image features of tumors provided an opportunity for the development of predictive biomarkers (32). Over the years, although several studies on the prediction performance of radiomics analysis to treatment response had been reported in patients with esophageal cancer, the predictive radiomics features were hard to reproduce since there were thousands of RFs and diverse selection criteria. For example, in the ^18^F-FDG PET, different radiomics features were found with discriminatory capability in predicting response to CRT in patients with esophageal cancer (33–35). Many researchers are committed to the standardization and unification of radiomics due to its virtue of convenience to use (36, 37). In our study, the radiomics signature was developed from 30 radiomics features from pretreatment images, and these radiomics features were selected with high interobserver reproducibility, high relevance, and low redundancy. Nevertheless, it was inadequate to predict the response merely using RFs from pretreatment images as what the single-shot image modeling did.

In our study, the selected features were extracted from a four-gray-level matrix, which was the ADC value matrix in the ADC map. Sixteen selected feature names were explained representing homogeneity or heterogeneity. The interpretation of other features was based on their calculation, including the distribution pattern of gray and the neighborhood intensity of the image. Their changes are recorded by ΔRFs. We proposed to train an SVM classifier by the RFs extracted from the images of four time points, including RF_pretreatment_, ΔRF_1st week_, ΔRF_2nd week_, and ΔRF_2 weeks_. R-Signature_pretreatment_ showed no correlation with treatment response. Instead, containing the change of whole-tumor heterogeneity over treatment, the radiomics signature based on ΔRF_2 weeks_ predicted the treatment response with an accuracy of 0.887, 0.826, and 0.765 in the three sets, respectively. In this study, the trend of ADC change during the initial two weeks showed an increase with no correlation with treatment response. This increase was thought to reflect the reduction in cell membrane integrity and changes in tumor cell numbers due to cytotoxic drugs and radiation, resulting in less restriction on the Brownion motion of water molecules (12). As the treatment progressed, tumor cells gradually decomposed and were absorbed, and the blood vessels and fibers in the microenvironment changed together. During this time, the changes of internal structures were not synchronized due to heterogeneity including different cell-cycle, oxygen status, and proliferative potential (38, 39). Our study was designed to record meaningful changes as early as possible (40). By screening radiomics features, effective predictions can be made through statistical methods. The results showed that the larger the time span, the more significant the changes obtained in radiomics features. Delta-radiomics has previously been used for the prediction of response to tumor treatment with ADC maps and CT (41, 42). As radiation and reaction to cytotoxic drugs accumulated, early changes from the tumor interior were recorded and quantified by a ratio that reduced the variability of the data. Moreover, Nasief et al. predicted the treatment response for CRT of pancreatic cancer by combining CT-based delta-radiomics and increasing CA19-9 (43).

In our study, esophageal tumor location was associated with treatment response in the training and external testing sets, while few studies had reported that the location of primary tumors was associated with the sensitivity of CRT in ESCC. One explanation was that biases existed in the clinical characteristic distribution in different responders with treatment due to the small sample size. Such as the result in the internal testing set, although the AUC changed, there was no increase in diagnostic performance. The other one was that the uneven dose distribution reduced the treatment effect since cervical esophagus had physiological curvature affected by the peripheral anatomical structure (44). The association of tumor locations and treatment response was to be further investigated in a larger cohort.

Our study had several limitations. Firstly, although an external testing set was included, the cohort of our study was still small. A multicenter study with a larger patient cohort is required. Secondly, though several selected procedures were used, esophageal tumors were manually delineated, which introduced an uncertain level of observer dependency. Thirdly, compared with RECIST 1.1 in the definitive cCRT, the pathological evaluation of neoadjuvant CRT was more convincing, and a systematic control comparison was supposed to be studied in the future.

In conclusion, we developed a radiomics signature-based model that incorporated the early change of RFs based on the sequential whole-tumor ADC map to predict treatment response to cCRT in patients with ESCC. This model provided a solution to quantify intratumoral changes and utilized them to guide treatment planning at an early date.

## Data Availability Statement

The raw data supporting the conclusions of this article will be made available by the authors, without undue reservation.

## Ethics Statement

The studies involving human participants were reviewed and approved by the Institutional Review Board of Shandong Cancer Hospital and Institute. The patients/participants provided their written informed consent to participate in this study. Written informed consent was obtained from the individual(s) for the publication of any potentially identifiable images or data included in this article.

## Author Contributions

Study conception and design: DA, BL, and QC. Collection of patient data: DA, YL, NS, and YZ. ROI delineation: WL and NS. Data analysis and statistical analysis: DA, QC, and ZL. Manuscript writing: DA. Manuscript editing: QC, ZL, and BL. All authors contributed to the article and approved the submitted version.

## Funding

This study has received funding from the National Natural Science Foundation of China (81530060), the National Natural Science Foundation of China (81874224), and the academic promotion program of Shandong First Medical University (2019LJ004).

## Conflict of Interest

The authors declare that the research was conducted in the absence of any commercial or financial relationships that could be construed as a potential conflict of interest.

## Publisher’s Note

All claims expressed in this article are solely those of the authors and do not necessarily represent those of their affiliated organizations, or those of the publisher, the editors and the reviewers. Any product that may be evaluated in this article, or claim that may be made by its manufacturer, is not guaranteed or endorsed by the publisher.
