# Supplementary figures and images for: Response Prediction to Concurrent Chemoradiotherapy in Esophageal Squamous Cell Carcinoma Using Delta-Radiomics Based on Sequential Whole-Tumor ADC Map
Source: Front Oncol. 2022 Mar 15;12:787489. doi: 10.3389/fonc.2022.787489 (PMC8982070; doi:10.3389/fonc.2022.787489)

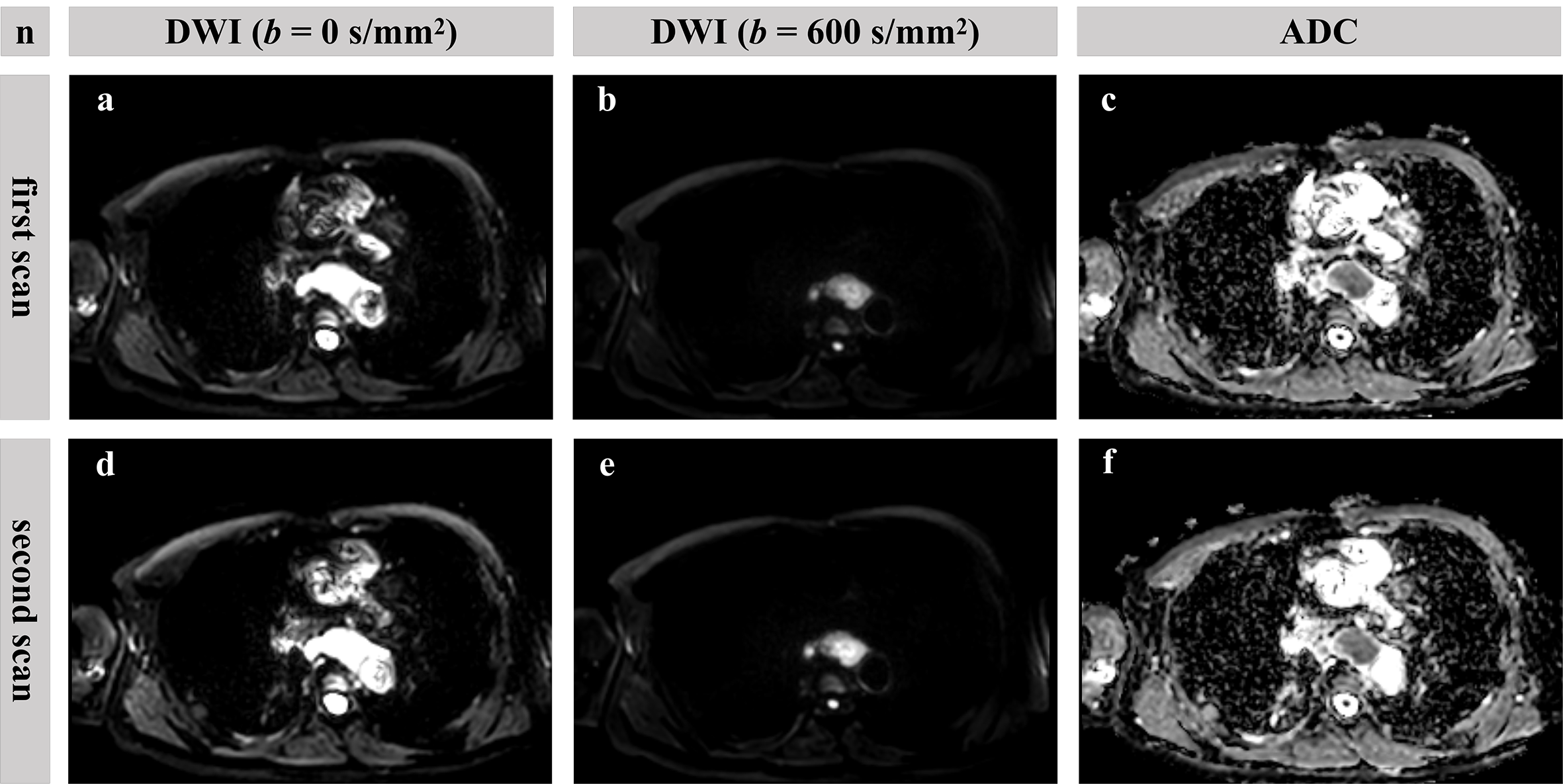

Supplement: Supplementary file 1 [file Image_1.tif]
