# Supplementary material for: Response Prediction to Concurrent Chemoradiotherapy in Esophageal Squamous Cell Carcinoma Using Delta-Radiomics Based on Sequential Whole-Tumor ADC Map
Source: Front Oncol. 2022 Mar 15;12:787489. doi: 10.3389/fonc.2022.787489 (PMC8982070; doi:10.3389/fonc.2022.787489)
Supplement: Supplementary file 4 [file Table_3.docx]

**Supplementary Table 3:** Association between voxel volume of ADC or the change of voxel volume and treatment response in the training, internal and external testing set.

| Set | Time point | voxel volume of ADC | | *p* | Time range | the change of voxel volume | | *p* |
| --- | --- | --- | --- | --- | --- | --- | --- | --- |
|  |  | Sensitive group | Resistant group |  |  | Sensitive group | Resistant group |  |
| Training  set | Pre-treatment | 18221±10827 | 15685±11783 | 0.253 | 1st  week | -0.146±0.291 | -0.011±0.391 | 0.322 |
|  | 5^th^ radiation | 14129±11818 | 15731±8663 | 0.269 | 2nd week | -0.053±0.146 | -0.035±0.170 | 0.717 |
|  | 10^th^ radiation | 11674±9024 | 11916±6591 | 0.568 | 2 weeks | -0.308±0.252 | -0.195±0.420 | 0.529 |
| Internal testing set | Pre-treatment | 21905±14053 | 12083±5392 | 0.130 | 1st  week | -0.180±0.352 | -0.082±0.365 | 0.363 |
|  | 5^th^ radiation | 16567±7490 | 11796±6407 | 0.080 | 2nd week | -0.030±0.120 | 0.081±0.630 | 0.857 |
|  | 10^th^ radiation | 12884±6570 | 8812±3876 | 0.257 | 2 weeks | 0.354±0.233 | 0.003±0.542 | 0.199 |
| External  testing set | Pre-treatment | 25249±15364 | 28387±9883 | 0.660 | 1st  week | -1.826±3.771 | -1.51±1.705 | 0.525 |
|  | 5^th^ radiation | 15068±10144 | 14825±7238 | 0.733 | 2nd week | -0.344±0.828 | 0.006±0.692 | 0.591 |
|  | 10^th^ radiation | 13145±8174 | 20821±11773 | 0.149 | 2 weeks | -1.327±1.712 | -0.607±0.724 | 0.404 |

Abbreviations: ADC, apparent diffusion coefficient.

**P* < 0.05, statistically significant.
