# Supplementary material for: Response Prediction to Concurrent Chemoradiotherapy in Esophageal Squamous Cell Carcinoma Using Delta-Radiomics Based on Sequential Whole-Tumor ADC Map
Source: Front Oncol. 2022 Mar 15;12:787489. doi: 10.3389/fonc.2022.787489 (PMC8982070; doi:10.3389/fonc.2022.787489)
Supplement: Supplementary file 5 [file Table_4.docx]

**Supplementary Table 4:** Association between maximum 3D diameter of whole-tumor or the change of maximum 3D diameter of whole-tumor and treatment response in the training, internal and external testing set.

| Set | Time point | maximum 3D diameter of whole-tumor | | *p* | Time range | the change of maximum 3D diameter of whole-tumor | | *p* |
| --- | --- | --- | --- | --- | --- | --- | --- | --- |
|  |  | Sensitive group | Resistant group |  |  | Sensitive group | Resistant group |  |
| Training set | Pre-treatment | 56.5±13.1 | 51.7±13.3 | 0.137 | 1st week | -0.034±0.154 | -0.011±0.145 | 0.819 |
|  | 5^th^ radiation | 49.8±14.2 | 55.0±11.7 | 0.132 | 2nd week | -0.161±0.219 | -0.176±0.287 | 0.819 |
|  | 10^th^ radiation | 48.2±11.1 | 51.0±11.5 | 0.303 | 2 weeks | -0.095±0.149 | -0.050±0.199 | 0.620 |
| Internal testing set | Pre-treatment | 60.7±13.5 | 52.0±9.37 | 0.199 | 1st week | -0.104±0.144 | -0.050±0.104 | 0.538 |
|  | 5^th^ radiation | 58.0±13.2 | 51.5±3.38 | 0.363 | 2nd week | -0.152±0.240 | 0.128±0.632 | 0.587 |
|  | 10^th^ radiation | 55.1±14.5 | 44.5±3.67 | 0.227 | 2 weeks | -0.144±0.099 | -0.002±0.253 | 0.150 |
| External testing set | Pre-treatment | 63.2±15.8 | 58.7±8.47 | 0.808 | 1st week | -0.286±0.634 | -0.121±0.141 | 1.000 |
|  | 5^th^ radiation | 54.8±17.1 | 52.3±2.87 | 0.808 | 2nd week | -0.038±0.295 | -0.016±0.231 | 0.884 |
|  | 10^th^ radiation | 53.6±10.6 | 53.7±10.2 | 0.961 | 2 weeks | -0.180±0.185 | -0.118±0.184 | 0.462 |

**P* < 0.05, statistically significant.
