# Supplementary material for: Response Prediction to Concurrent Chemoradiotherapy in Esophageal Squamous Cell Carcinoma Using Delta-Radiomics Based on Sequential Whole-Tumor ADC Map
Source: Front Oncol. 2022 Mar 15;12:787489. doi: 10.3389/fonc.2022.787489 (PMC8982070; doi:10.3389/fonc.2022.787489)
Supplement: Supplementary file 9 [file Table_8.docx]

**Supplementary Table 8:** Univariate logistic regression analysis of primary tumor site and radiomics

signture_2 weeks_ in the training set.

| Model | Coefficient | *p* | OR | lower 95%CI | upper 95%CI |
| --- | --- | --- | --- | --- | --- |
| Tumor location | 0.750 | 0.011 | 2.435 | 0.294 | 6.501 |
| Signture_2 weeks_ | 4.626 | <0.0001 | 65.340 | 8.995 | 325.565 |

Abbreviations: OR, odd ratio; CI, confidence interval.

**P* < 0.05, statistically significant.
