# Supplementary material for: Response Prediction to Concurrent Chemoradiotherapy in Esophageal Squamous Cell Carcinoma Using Delta-Radiomics Based on Sequential Whole-Tumor ADC Map
Source: Front Oncol. 2022 Mar 15;12:787489. doi: 10.3389/fonc.2022.787489 (PMC8982070; doi:10.3389/fonc.2022.787489)
Supplement: Supplementary file 10 [file Table_9.docx]

**Supplementary Table 9:** Multivariate logistic regression analysis of clinical characteristics and signture_2 weeks_ in the training, internal and external testing set.

| Set | Variable | Coefficient | *p* | OR | lower 95%CI | upper 95%CI |
| --- | --- | --- | --- | --- | --- | --- |
| Training set | Tumor location | 4.712 | 0.018 | 4.895 | 0.898 | 52.358 |
|  | R-Signture_2 weeks_ | 6.231 | <0.0001 | 189.344 | 26.584 | 1623.812 |
| Internal testing set | Tumor location | -- | 0.423 | -- | -- | -- |
|  | R-Signture_2 weeks_ | 2.485 | 0.035 | 12.000 | 2.185 | 121.570 |
| External testing set | Tumor location | 2.015 | 0.041 | 1.600 | 0.104 | 2.567 |
|  | R-Signture_2 weeks_ | 2.197 | 0.039 | 9.000 | 1.915 | 88.576 |

Abbreviations: OR, odd ratio; CI, confidence interval.

**P* < 0.05, statistically significant.
